# Application Deadline for The CDC Experience Applied Epidemiology Fellowship — December 6, 2013

**Published:** 2013-10-04

The CDC Experience is a 1-year fellowship in applied epidemiology for third- and fourth-year medical students. Eight competitively selected fellows spend 10–12 months at CDC in Atlanta, Georgia, where they conduct epidemiologic analyses in areas of public health that interest them. The fellowship provides opportunities to enhance skills in research and analytic thinking, written and oral scientific presentations, and the practices of preventive medicine and public health.

Through this training, fellows acquire practical knowledge for approaching population-based health problems. Graduates of The CDC Experience have an appreciation of the role of epidemiology in medicine and health and are able to apply their knowledge and skills to enhance their clinical acumen and help improve the quality of the U.S. health-care system.

Information on applying for The CDC Experience is available at http://www.cdc.gov/cdcexperiencefellowship. Applications for the class of 2014–15 must be submitted by December 6, 2013. Questions can be e-mailed to Virginia Watson, program coordinator, at vwatson1@cdc.gov.

